# DVL1 and DVL3 differentially localize to CYP19A1 promoters and regulate aromatase mRNA in breast cancer cells

**DOI:** 10.18632/oncotarget.26257

**Published:** 2018-11-02

**Authors:** Isabel Castro-Piedras, Monica Sharma, Meghan den Bakker, Deborah Molehin, Edgar G. Martinez, David Vartak, Wendy M. Pruitt, Jena Deitrick, Sharilyn Almodovar, Kevin Pruitt

**Affiliations:** ^1^ Department of Immunology & Molecular Microbiology, Texas Tech University Health Sciences Center, Lubbock, TX, USA

**Keywords:** aromatase, CYP19A1, dishevelled, DVL, breast cancer

## Abstract

The CYP19A1 gene encodes aromatase, an enzyme that converts androgens into estrogens and consequently directly contributes to both the depletion of androgens and the synthesis of estrogens in several organs. Aromatase is critical for diverse biological processes such as proliferation, regulation of fat metabolism and hormone signaling. Additionally, it is also overexpressed in diverse cancers and drives hormone-dependent tumor progression and increases 17-β-estradiol (E_2_) within tumors and the tumor microenvironment. Although the inhibition of E_2_ production via aromatase inhibitors represents a major therapeutic paradigm in clinical oncology, fundamental questions regarding how cancer cells gain the capacity to overexpress aromatase remain unanswered. Multiple tissue-specific CYP19A1 promoters are known to be aberrantly active in tumors, yet how this occurs is unclear. Here, for the first time, we report that Dishevelled (DVL) proteins, which are key mediators of Wnt signaling, regulate aromatase expression in multiple breast cancer cell lines. We also report that DVL enters the nucleus and localizes to at least two different CYP19A1 promoters (pII and I.4) previously reported to drive overexpression in breast tumors and to a very distal CYP19A1 placental promoter (I.1) that remains poorly characterized. We go on to demonstrate that DVL-1 and DVL-3 loss of function leads to differential changes in various aromatase transcripts and in E_2_ production. The report, herein, uncovers a new regulator of CYP19A1 transcription and for the first time demonstrates that DVL, a critical mediator of WNT signaling, contributes to aberrant breast cancer-associated estrogen production.

## INTRODUCTION

While studies report that DVL function is altered in diverse pathophysiological settings, its mechanistic role remains unclear in many of these conditions [1–3]. WNT signaling is critical for organismal development and DVL integrates an immense number of upstream signals that may arise from as many as 19 different WNTs, 10 Frizzled receptors and multiple co-receptors and secreted antagonists. The complex oncogenic signals produce potent stimuli which are relayed by DVL as it serves as a critical hub for transmitting these cues [1]. Despite its central role in WNT signaling and development, DVL has never been linked with any aspect of steroidogenesis. Historically, DVL has been studied almost exclusively in the context of its cytosolic role of promoting β-catenin stabilization [4] or cell migration [5, 6]. However, recent studies show DVL translocation to the nucleus and binds the promoters of a limited number of WNT target genes such as cMyc, BMP4, cyclin D1 [7] and FZD7 [8]. Moreover, FOXK transcription factors were reported to associate with DVL and facilitate its nuclear translocation, a process that is important for Wnt/β-catenin signaling [9]. The process of nuclear translocation is proving significant so efforts to identify the genes bound by DVL may clarify how constitutive Wnt signaling contributes to various stages of tumorigenesis or congenital diseases. While chronic WNT pathway activation in colorectal cancers is mostly driven by gene mutations [10], epigenetic changes largely act as a driver in breast cancer [11–13]. Sustained WNT pathway activation is frequent in specific breast cancer subtypes and alters many WNT target genes that can be either silenced in the case of some tumors suppressor genes [14–17] or activated as with some oncogenes [18, 19]. We set out to screen for novel target genes of DVL given its role in promoting oncogenesis [5, 20–22]. Previous reports have demonstrated that DVLs function cooperatively, as well as uniquely, in the mediation of Wnt3a-stimulated canonical signaling [23] and with respect to the role of conserved domains [24]. This concept that DVL isoforms in mammals may operate as a network in some cellular contexts yet exhibit specificity in other cellular contexts is supported by our findings. The study herein reports, for the first time, a novel link between different DVL protein family members and their role as regulators of multiple tissue-specific CYP19A1 transcripts that are aberrantly expressed in tumors.

The CYP19A1 gene encodes the aromatase enzyme that converts androgens into estrogens and consequently profoundly contributes to diverse biological processes. We now know that aromatase is elevated in diverse cancers and drives hormone-dependent breast and endometrial tumor progression. Early on, studies demonstrated a significant correlation between aromatase activity and tumor incidence in individual quadrants of breast tissue [25] and highlighted the need to identify factors that regulate steroid metabolism in peripheral tissue and tumors [26]. Further investigation demonstrated that aromatase mRNA and protein was not only detected in human breast tumors [27, 28], but was increased in breast tumor tissue relative to non-malignant breast tissue [29] or relative to matched non-neoplastic cells [30, 31]. Aromatase is elevated in most breast cancers [32] and interestingly in other cancers not typically associated with dysregulated steroidogenesis such as NSCLC [33] and colon cancer [34]. Aromatase has even been linked with metastasis [35, 36] and transgenic models demonstrate its oncogenicity [37, 38], yet many unknowns remain about the diverse pathway(s) that drive CYP19A1 overexpression. This question is important because tumor-associated estrogen production was shown to be significantly higher than levels in the plasma of post-menopausal patients or in normal tissue [39]. Additionally, high estrogen levels in the tumor microenvironment skew the ratio of effector to regulatory T cells, and high estrogen promotes expansion and recruitment of Tregs which severely dampens the ability of the immune system to fight and destroy tumor cells [40, 41]. Although aromatase inhibitors (AI) represent a major clinical therapeutic strategy for cancers and certain developmental disorders, there are many gaps in knowledge regarding the factors that drive the dysregulated expression of the aromatase tissue-specific transcripts in breast cancers. Adding to the complexity, studies show that two tightly linked SNPs in the placental promoter significantly predict aromatase activity and patients show higher plasma E_2_ during pre-AI and post-AI treatment [42]. Here, we report for the first time that DVL proteins localize to multiple CYP19A1 tissue-specific promoters, including the ovary, skin/adipose tissue and placental promoters. We demonstrate that specific DVLs have overlapping and distinct roles with respect to regulation of binding CYP19A1 tissue-specific promoters and regulating the expression of promoter-specific CYP19A1 transcripts. These findings uncover a new regulator of CYP19A1 mRNA which may play a role in contributing to the aberrant tumor-associated estrogen production.

## RESULTS

### Breast cancer cells express multiple aromatase mRNA transcripts

**Figure 1:**
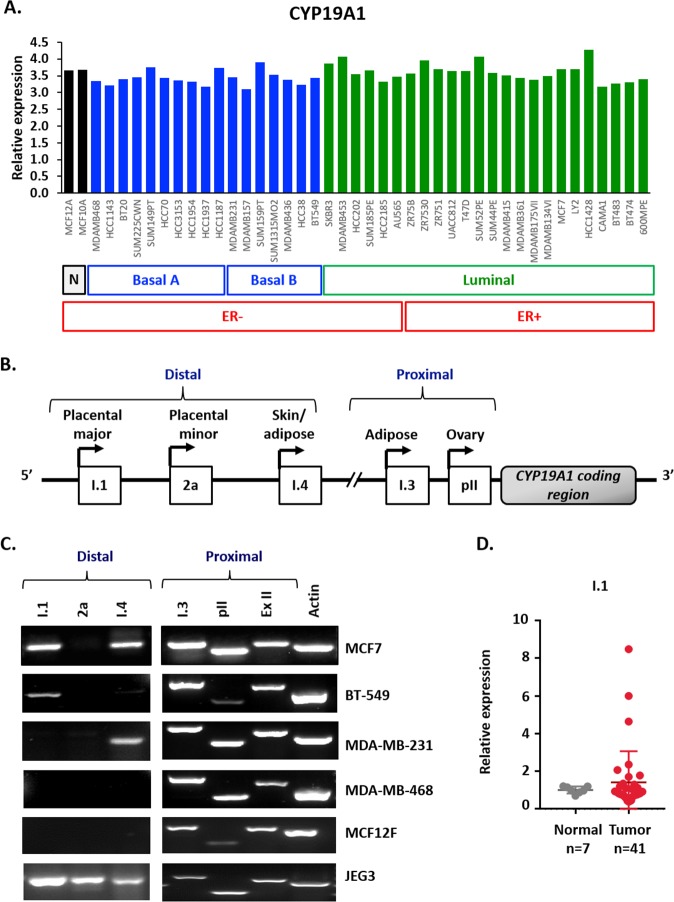
Multiple aromatase transcripts are expressed in multiple cancer cell lines, and the placental aromatase transcript is expressed in breast cancer tissues **(A)** Relative aromatase RNA expression in breast cancer cell lines using Heiser RNASeq data downloaded from UCSC Xena platform. **(B)** Schematic representation of the different tissue-specific CYP19A1 promoters: I.1, 2a, I.4, I.3 and pII. **(C)** Identification of aromatase active promoters in breast cancer cell lines (MCF7, MDA-MB-231, BT-549 and MDA-MB-468), non-cancer breast cell line (MCF12F) and placental cell line (JEG3) by end-point PCR. Amplification of three distal promoters, between 93 to 73Kb from ATG: placental aromatase transcripts (I.1, 2a) and skin/ adipose tissue transcript (I.4), two proximal, 0.2 Kb from ATG or less: adipose/breast cancer (I.3) and ovary/breast cancer (pII), exon II (ExII) common in all aromatase transcripts and beta-actin as control. **(D)** Expression of I.1 promoter in breast cancer TissueScan array (Origene) by real time qPCR, represented as average fold change of I.1 promoter respect to beta-actin and normalized to normal tissue.

We previously reported that sirtuin-1 (SIRT1), β-catenin, and DVL partner in the regulation of Wnt signaling at multiple levels [5, 22]. We also found that SIRT1 positively regulates aromatase transcription in breast cancer cells and binds the I.3/pII and pI.4 promoter regions [43]. Based on our previous findings, we set out to determine if there was a connection between DVL and aromatase. To determine whether DVL is connected with CYP19A1, we first wanted to establish which breast cancer cell lines express aromatase mRNA. While the relative levels to total aromatase mRNA were found to vary across 43 breast cancer lines, each expressed aromatase mRNA independently of ER-status or subtype (Figure 1A) [44]. We next examined the expression of tissue-specific transcripts shown in a schematic form (Figure 1B) across a panel of four breast cancer, one placental choriocarcinoma and one non-cancer breast epithelial cell lines. We performed 5′-UTR-specific RT-PCR using a forward primer that is specific to the unique 5′UTR and a reverse primer against a common protein coding exon (Figure 1C). This enables detection of various aromatase mRNAs that contain an alternative noncoding exon 1 that precedes a common protein-coding region. RNA was isolated from four breast cancer cell lines (MCF7, MDA-MB-231, BT-549, MDA-MB-468), one placental choriocarcinoma line (JEG3) and one non-cancer breast epithelial line (MCF12F). These cell lines represent different breast cancer subtypes and we previously demonstrated that WNT signaling is important in mediating oncogenic signaling across the different subtypes [8, 22, 45]. We found that ovary (pII) and adipose (I.3) transcripts (Figure 1C), both of which are frequently upregulated in breast cancer, were expressed in all cell lines. This prevalence of pII and pI.3 mRNA cancer-associated expression is consistent with previous reports [27, 29, 46]. Moreover, we found the skin/adipose transcript (I.4) (Figure 1C) and the placenta major transcript (I.1) (Figure 1C and Supplementary Figure 1A) were expressed in three of the five cancer cell lines. In contrast, the placenta minor (2a) transcript was only observed in the JEG3 cells while none of the transcripts associated with the distal alternative first exons were expressed in MCF12F non-cancer cells. The I.1 transcript is the most distal from the protein coding exon II that lies 90kb away from the non-coding I.1 exon (Figure 1B). Although some reports indicate that the I.1 transcript is expressed in tumors [33] and select cell lines [27, 47], the majority of previous reports focused more on the I.3 and pII promoters and demonstrated their increased expression in primary breast tumors. Thus, we wanted to further explore and validate the expression of I.1. To further validate expression of I.1 we performed 5′UTR-specific RT-PCR using cDNA generated three different ways from MCF7 mRNA and indeed observed its expression (Supplementary Figure 1A). To further characterize the I.1 promoter, we performed chromatin immunoprecipitation (ChIP) for an active (H3K4me3) and repressive (H3K27me3) histone mark at multiple regions along the I.1 promoter. We found that the H3K4me3 mark, which correlates with open transcriptionally permissive chromatin, is enriched across the I.1 promoter in MCF7 cells. We performed 8 independent H3K4 and H3K27 ChIP experiments at multiple regions of the I.1 promoter and two representative experiments are shown in (Supplementary Figure 1B). Together, these findings demonstrate that multiple breast cancer lines express multiple aromatase transcripts, including the poorly characterized I.1 transcript. We further evaluated the expression of the I.1 transcript in breast cancer tissues using a breast cancer cDNA array. We found that the I.1 promoter was expressed in the 17% of the breast cancer specimens (n=41) but was not expressed in any of the controls (n=7) (Figure 1D). The cases in which I.1 was expressed spanned the spectrum and included ER+/PR+, ER+/PR+/HER2+, and triple negative cases. These findings demonstrate that the I.1 transcript is not only expressed in breast cancer cell lines but is also expressed in primary breast tumors.

### DVL proteins are present in the nucleus and cytoplasm of breast cancer cells expressing multiple aromatase transcripts

**Figure 2:**
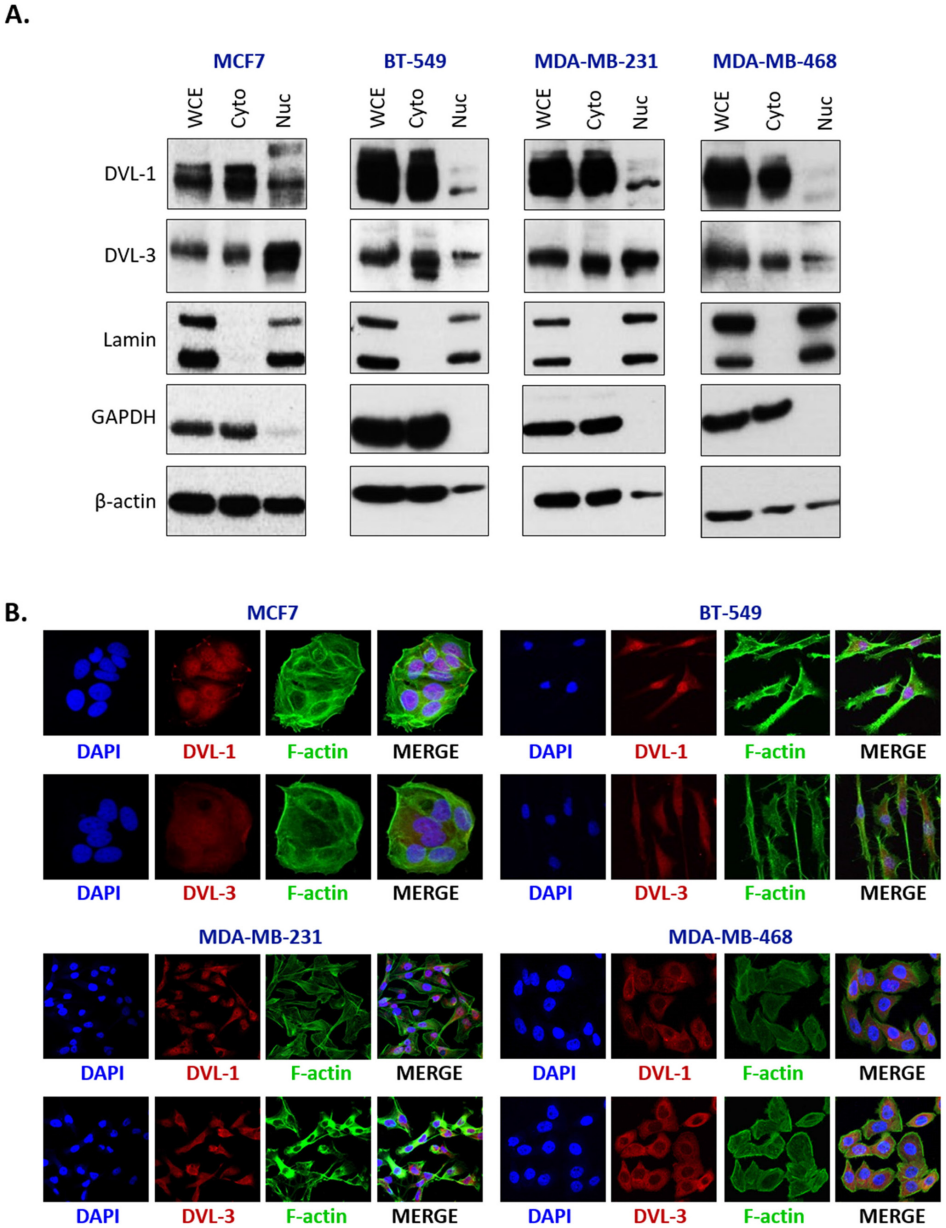
DVL proteins are localized in the nucleus and cytoplasm of different breast cancer cells **(A)** Nuclear and cytoplasmic extracts from four breast cancer cells (MCF7, BT-549, MDA-MB-231, and MDA-MB-468) were analyzed using Western blots. The blots were probed with DVL-1 and DVL-3 antibodies. Lamin was used as a control for nuclear extract and GAPDH was used as a control for cytosolic proteins. **(B)** Immunofluorescence was performed to analyze DVL proteins localization in MCF7, BT-549, MDA-MB-231, and MDA-MB-468 cells. The cells were probed with DVL-1 and DVL-3 antibodies (red). The nucleus was stained with DAPI (blue) and the actin filaments (green) were stained with Phalloidin.

Since there are limited reports suggesting that DVL undergoes nuclear localization and little is known about whether it binds to gene promoters, we wanted to further investigate this angle and prepare for DVL ChIP analyses by determining which DVL proteins translocate to the nucleus and if this occurs across multiple breast cancer lines. Given our previous findings demonstrating that DVL-1 plays a critical role in regulating a TIAM1-Rac1 signaling axis in MDA-MB-231 cells [5], and endogenous DVL-3 co-precipitates with SIRT1 in breast cancer cells [22], we were interested in exploring these two family members in particular. We analyzed the cytosolic vs. nuclear distribution of DVL across four breast cancer lines (MCF7, BT-549, MDA-MB-231, and MDA-MB-468). Following nuclear-cytoplasmic fractionation, protein extracts were analyzed via Western blotting. Interestingly, DVL-1 and DVL-3 were present at different levels in both nuclear and cytoplasmic fractions in multiple breast cancer lines (Figure 2A). To further investigate subcellular localization, immunofluorescence (IF) was performed across all four breast cancer cell lines. Consistent with the fractionation experiments we found that DVL proteins show nuclear localization to varying degrees in multiple cell lines via IF (Figure 2B). Overall, the results demonstrate that endogenous DVL proteins localize to both the nucleus and cytoplasm across multiple breast cancer cell lines.

### DVL binds multiple CYP19A1 tissue-specific promoters

**Figure 3:**
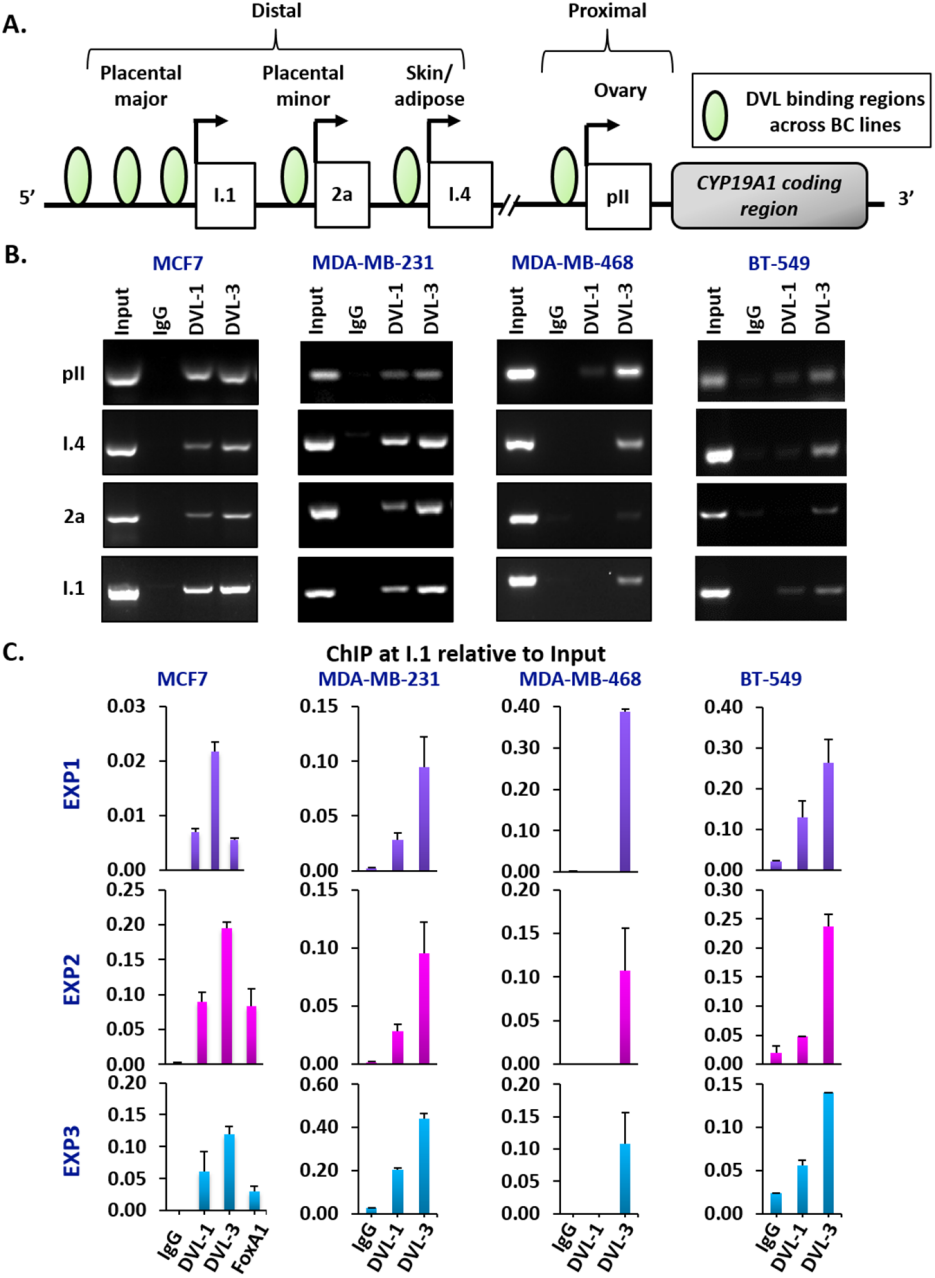
DVL family members bind to multiple CYP19A1 promoters **(A)** Schematic representation of different tissue-specific CYP19A1 promoters located proximally (pII, and I.3) or distally (I.4, 2a, and I.1) with respect to the coding region. The green circles represent the genomic region bound by DVL proteins in different breast cancer cells **(B)** Three independent ChIP experiments for IgG, DVL-1 and DVL-3 were performed in MCF7, MDA-MB-231, MDA-MB-468 and BT-549 cells. Occupancy of DVL at four tissue-specific promoters of CYP19A1 gene (pII, I.4, 2a, & I.1) were analyzed by end-point PCR. **(C)** Three independent ChIP-qPCR experiments at I.1 promoter for IgG, DVL-1, DVL-3 and FOXA1 were performed in MCF7 and for IgG, DVL-1 and DVL-3 in MDA-MB-231, MDA-MB-468 and BT-549 cells. error bars = std dev of triplicates.

After establishing that DVL proteins are present in the nucleus, we next wanted to determine whether one of the family members might bind to CYP19A1 promoters given that its binding partners (SIRT1 and β-catenin) had been shown to occupy specific promoters [43, 48]. We designed chromatin immunoprecipitation (ChIP) primers that spanned about a 1kb region relative to the TSS of multiple aromatase promoters as shown in Figure 3A. We performed ChIP-PCR for DVL-1 and DVL-3 in MCF7 and MDA-MB-231 cells and found that both occupy the pII and pI.4 promoters, both of which are widely shown to be active in breast cancers (Figure 3B). We also found DVL-3 at the pII and pI.4 promoters in MDA-MB-468 and BT-549 cells. We further found that both DVLs bind the 2a promoter in MCF7 and MDA-MB-231 cells, but only DVL-3 appears to bind the 2a promoter in MDA-468 and BT-549 cells. In addition, we found both DVLs occupy the I.1 promoter in MCF-7, MDA-MB-231 and BT-549 cells, but only DVL-3 appears to bind the I.1 promoter in MDA-MB-468 cells (Figure 3B and Supplementary Figure 2). Interestingly, these results demonstrate that DVL occupies both active and inactive CYP19A1 promoters. Because DVL is known to scaffold other proteins, we reasoned that it could be helping to activate or repress in a promoter-specific manner. We further investigated DVL binding to the I.1 promoter since it is less well-characterized. We performed ChIP for DVL-1, DVL-3 and FOXA1 coupled with quantitative PCR (ChIP-qPCR) given previous analysis of FOXA1 ChIPseq in MCF7 cells [49]. As shown in three independent experiments in a panel of breast cancer cell lines (Figure 3C), we found that both DVL-1 and DVL-3 bind the I.1 promoter in MCF7, MDA-MB-231 and BT-549 while only DVL-3 binds to I.1 in MDA-MB-468. (Figure 3C).

### DVL family members regulate the levels of multiple tissue-specific aromatase transcripts, estrogen production, and cell

**Figure 4:**
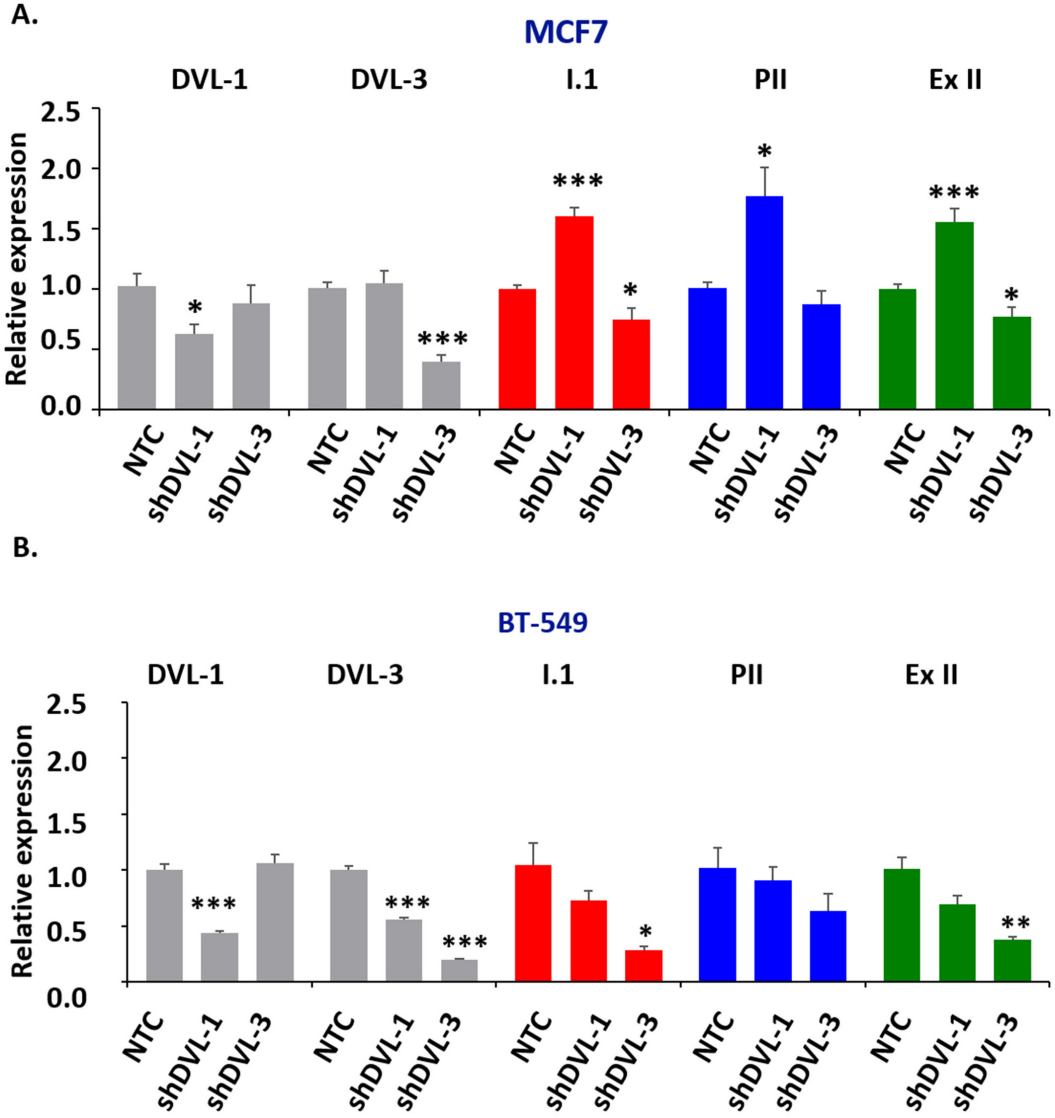
DVL loss of function alters aromatase transcript levels **(A)** RNA isolated from MCF7 and BT-549 cells stably expressing a non-targeting control shRNA (NTC), a DVL-1 shRNA or DVL-3 shRNA was converted to cDNA. Quantitative PCR was then performed using primers specific for DVL-1 (panel 1), DVL-3 (panel 2), the placental I.1 aromatase transcript (panel 3), the ovary PII aromatase transcript (panel 4) or the total aromatase mRNA with primers in the coding region common to all transcripts (panel 5). **(B)** RNA isolated from BT-549 cells and analyzed as described in (A). Data represent fold change respect to beta-actin, performed in triplicate with values as mean ± SEM, n=3 and normalized to NTC cells, p-values correspond to ^*^ p<0.05, ^**^ p<0.01, ^***^ p<0.001.

**Figure 5:**
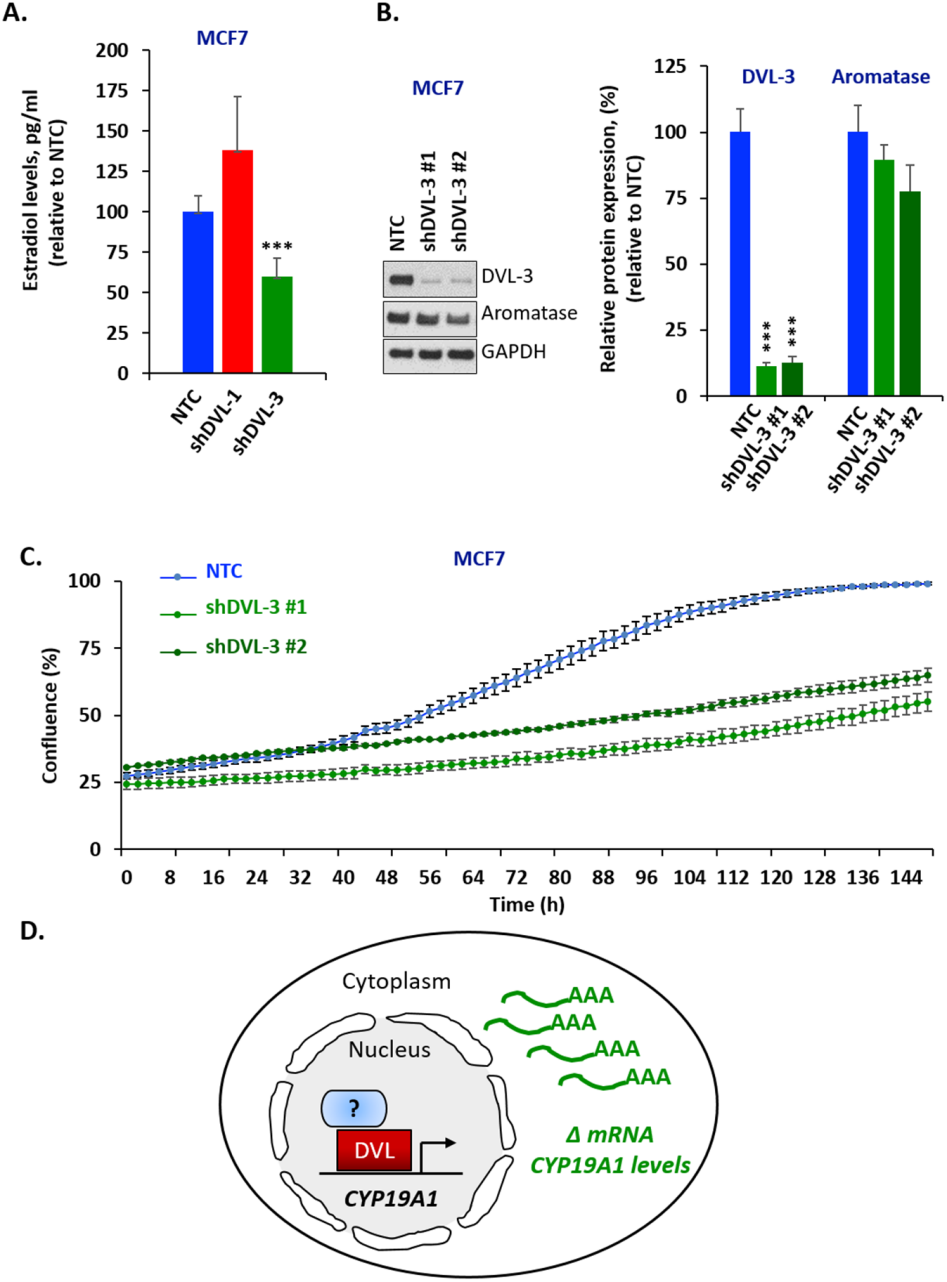
DVL loss of function alters estrogen levels and cell proliferation **(A)** Estradiol levels of MCF7 cells expressing stable knockdown of DVL-1 (shDVL-1) and DVL-3 (shDVL-3) and non-target control (NTC) treated with 10nM androstenedione for two days. Data are representative of 5 independent experiments carried out in triplicate with std dev, ^**^, *p*= 0.0008. **(B)** Whole cell extracts from MCF7 NTC, MCF7 shDVL-3 #1 and MCF7 shDVL-3 #2 where analyzed using Western blots. The blots were probed with DVL-3, aromatase and GAPDH antibodies. **(C)** Time course of growth curve of MCF7 cells expressing stable knockdown of DVL-3 (shDVL-3 #1 and shDVL3 #2) and non-target control (NTC) cell proliferation was measured as percent confluence from phase-contrast images. Plot shows mean and SEM. Data are representative of 3 independent experiments carried out in octuplicate, ^***^ p<0.001 after 70 h. **(D)** Schematic representation of DVL proteins binding to CYP19A1 promoter region and regulating its mRNA level.

DVLs are known to hetero-dimerize and form oligomeric structures in different contexts, so we next wanted to determine whether DVL-1 or DVL-3 loss of function would change I.1 transcript expression. We stably knocked-down DVL-1 and DVL-3 individually in MCF7 and BT-549 cells considering both cell lines had active pII and I.1 promoters, to study how loss of function of each DVL would affect the pII and I.1 transcripts. These two transcripts were chosen because they have been shown to correlate the best with aromatase protein expression [50]. We used 5′-UTR-specific real-time qPCR with β-actin as internal control to determine if DVL loss of function altered either the PII or I.1 transcripts or total aromatase mRNA. Interestingly, we found that stably knocking down DVL-1 in MCF7 cells caused a statistically significant increase in the PII, I.1 and total (Ex-II) aromatase transcripts while DVL-3 knockdown caused a statistically significant decrease in the I.1 and total aromatase transcripts (Figure 4A). Furthermore, we found a different trend in BT-549 cells. We observed that when DVL-1 was depleted, this led to a statistically significant decrease in DVL-3 mRNA (Figure 4B). However, depletion of DVL-1 did not cause a statistically significant reduction in the levels of the PII, I.1 and total aromatase transcripts. In contrast, in both MCF7 and BT-549 cells, we found that DVL-3 depletion caused a statistically significant decrease in the I.1 and total aromatase transcripts (Figure 4B). Overall, these results show that DVL proteins not only bind multiple aromatase promoters, but they differentially regulate tissue-specific aromatase transcripts in both ER+ (MCF7) and ER- (BT-549) cells. Next, to determine whether depletion of DVL-1 vs. DVL-3 in MCF7 cells could alter the production of E2, we performed ELISA analyses. Relative to the non-targeting control (NTC), stable knockdown of DVL-1 in MCF7 cells showed a trend of higher E_2_ production, although this did not reach statistical significance. Conversely, knockdown of DVL-3 caused a statistically significant reduction in E_2_ by approximately 50% (Figure 5A). Aromatase protein levels were evaluated in cells in which DVL-3 was stably knocked down with 2 different shRNAs. With the extent of knockdown of DVL-3 alone, we observed a trend towards reduced protein levels with both constructs (Figure 5B). Subsequently, we examined if the reduction in E_2_ production, due to DVL-3 knockdown, also changed cell proliferation *in vitro* by real-time imaging. This evidence indicates that stable downregulation of DVL-3 significantly reduced cell proliferation in comparison to NTC in MCF7 cells (Figure 5C). Together, these data demonstrate that DVL proteins serve as regulators of aromatase. Not only do DVLs bind to multiple tissue-specific aromatase promoters that are aberrantly activated in cancer, but the role of DVL-1 vs. DVL-3 appears to play a promoter-specific and cell- type dependent role that can lead to either activation or repression of CYP19A1 transcripts (Figure 5D).

## DISCUSSION

Aromatase overexpression is found in the majority of breast cancers and leads to chronic intra-tumoral increase in estrogens [51, 52]. In tumors, CYP19A1 transcription is driven by multiple promoters that somehow override the tissue-specific regulation characteristic of normal tissue [53, 54]. While much progress has been made describing the active promoters in cancer [55], many unknowns remain regarding the factors that promote aberrant CYP19A1, especially for transcription associated with the more distal alternative exons such as I.1. Tissue-specific regulation of aromatase is critical as this provides a local source of estrogens which influences growth, survival or hormone-dependent signaling that can be uncoupled from the ovarian cycle. Additionally, this tissue-specific production of estrogen also ensures that, during the post-menopausal years, the tissues and cells still requiring estrogen for non-reproductive functions will retain this capacity as the ovarian source of estrogen subsides. Because estrogens promote growth and proliferation, neoplastic cells very frequently exploit this property and aromatase is frequently elevated in tumors. Recently, we reported that the aromatase protein is subject to novel post-translational regulation which provides a more rapid modulation of its enzymatic activity [56]. Aromatase post-translational regulation such as lysine acetylation which we recently demonstrated in different domains [56] may influence aromatase antibody affinity if the epitope undergoes post-translational modifications (PTM). Some of the aromatase antibodies used in early studies (such as MCA2077) were generated against epitopes (such as aa 376-390) that we recently demonstrated undergo post-translational modification in MCF7 cells. We recently demonstrated via LC-MS/MS that at least two of the three lysines (K376 & K390) within this antigenic peptide undergo lysine acetylation. Because of these PTMs, it will be important for future studies to compare the mRNA and protein levels across cell lines and cancer tissues with more recent aromatase antibodies. Studies have shown that overexpression of aromatase in mammary tissue in transgenic animals is sufficient for maintenance of hyperplasia in the absence of circulating estrogens. Also, aromatase inhibitors abrogate the aromatase-induced hyperplasia which further demonstrates the potency of increased levels of local estrogens [38]. Aromatase overexpression in mice was also shown to induce pre-neoplasia and carcinoma formation. While this study demonstrated that increased expression of both ERα and aromatase activate abnormal growth pathways in the mammary gland, aromatase was shown to induce a wider range of abnormalities that was associated with a higher prevalence of mammary pre-neoplasia and cancer progression [57]. Other reports have shown that aromatase overexpression in ERα-negative benign cells triggers numerous hallmarks of cancer and induces tumor-promoting estrogen metabolites that damage DNA [58, 59]. Because aromatase contributes to multiple facets of tumor progression and clinical trials demonstrate higher benefit in patients treated with aromatase inhibitors compared to tamoxifen [60], recommendations have been made to incorporate an AI to reduce the risk of breast cancer recurrence [61]. However, what causes the frequent and sustained upregulation of aromatase across diverse tumors remains unclear. Our findings here provide additional insight into its transcriptional regulation and for the first time implicate DVL in this process.

Aberrant changes in transcriptional regulatory regions contribute to cancer initiation and progression [62]. One example of this occurs during “promoter switching” which contributes to abnormal CYP19A1 gene activation. Promoter switching causes a disproportional use of some CYP19A1 promoters (such as promoters pI.3, pI.7 and pII) instead of the exclusive use of breast/adipose promoter I.4 typically activated in normal breast tissue [51]. Interestingly, while adipose (pI.3), skin/adipose (pI.4), endothelial (pI.7) and ovary (pII) were shown to be activated during cancer progression [36, 55, 63], another report demonstrated that the transcripts associated with pI.1 and pII were shown to best correlate with aromatase protein expression [50]. This study further highlighted the concept that the 5′UTR encoded in the alternative exon I is subject to posttranscriptional regulation that directly impacts protein abundance. Considering this, of all 11 unique CYP19A1 promoters that could drive aromatase production, the transcripts associated with the placental (pI.1) and ovary (pII) promoters would be predicted to yield the highest level of aromatase protein [50]. Some factors have been implicated in the regulation of I.1 [64]; however, factors that drive transcription from I.1 in tumors and the mechanism driving promoter switching are poorly understood. Remarkably, our study shows that the knockdown of DVL-3 leads to i) reduced levels of the I.1 and total aromatase transcripts ii) decreased E_2_ production and iii) decreased proliferation in MCF7 cells. Interestingly, the DVL-1 binding pattern may be cell line specific as the knockdown of DVL-1 increases the expression levels of I.1, pII and total aromatase transcripts in MCF7 cells, but not in BT-549 cells. While pII promoter has received much of the focus in breast cancers, recent studies suggest that the I.1 promoter contributes to pathology. One report from Wang et al [42] helped to better establish a connection between I.1 and breast cancer. In this study, two tightly linked SNPs in the 5′-flanking region of CYP19A1 exon I.1 were significantly associated with a greater change in aromatase activity after AI treatment. Additionally, these same two SNPs were also associated with higher plasma E_2_ levels in patients during pre-AI and post-AI treatment [42]. Interestingly, we find that the region of DVL binding identified in ChIP encompasses these upstream regions where one of the SNPs (rs7176005) is located. Very limited analysis of the I.1 promoter and its regulation has been performed. One report demonstrated that a I.1 luciferase promoter construct containing a −924 bp region upstream of the TSS of exon I.1 showed the highest induction by serum in JEG3 cells [65]. Another study demonstrated that a placenta promoter variant −41 base pairs upstream of exon I.1 resulted in a significantly reduced transactivation ability of 50% compared to wild-type [66]. Interestingly, this study demonstrated that while the ovarian promoter was normal in the patient described in the report, her placenta promoter carried a heterozygote single C > T base exchange at position −41bp from exon I.1. Of the 100 controls analyzed, this substitution was present in 21% of the controls, yet the functional consequences have not been explored. Overall, we now know that DVL binds to multiple CYP19A1 promoters and influences the levels of multiple aromatase transcripts. The study herein, for the first time, reports a novel link between DVL proteins and their role as regulators of transcription at aromatase promoters. This report further clarifies another aspect of the nuclear role of DVL and its link with CYP19A1 regulation which has never been reported. These findings provide deeper insight into a major oncogenic pathway that may be involved in promoter switching.

## MATERIALS AND METHODS

### RNA analysis *in silico*

Relative RNA expression of CYP19A1 gene in breast cancer cell lines was downloaded from UCSC Xena plataform on 12th of August 2018 from Heiser RNASeq data (18632 genes in 54 breast cancer cell lines) [44].

### Cell lines

All the cell lines (MCF7, MDA-MB-231, BT-549, MDA-MB-468, MCF12F and JEG3) used in this manuscript were purchased from ATCC which utilizes STR technology for Cell Authentication, and they were used in a low passage (<20) within 6 months or less after receipt or resuscitation. MDA-MB-231 and JEG3 cells were cultured in D-MEM (Gibco) supplemented with Na Pyruvate (Sigma), MCF7 cells were cultured in MEM (Gibco) supplemented with 0.1% insulin (Sigma), MCF12F cells were cultured in HuMEC Basal Serum Free Medium (Gibco) supplemented with HuMEC supplement kit (Gibco) while BT-549 and MDA-MB-468 cells were cultured in RPMI 1640 (Gibco) supplemented with 0.1% insulin. All culture media were supplemented with 10% fetal bovine serum and 1% penicillin/streptomycin (Invitrogen) except MCF12F cells.

### Expression analysis

Total RNA was isolated from cancer cell lines as well as DVL stable knock-down cells using Pure-link RNA mini kit (Invitrogen). 2μg of total RNA was reverse-transcribed using SuperScript III Reverse Transcriptase (ThermoFisher) to synthesize first-strand of complementary DNA (cDNA), using gene specific reverse primers for beta-actin (GSP-Actin) and aromatase (GSP-Aromatase) for all the aromatase transcripts and using an Oligo(dT)20 Primer (ThermoFisher) for the DVL transcripts.

Human breast cancer cDNA array was purchased from ORIGENE (BCRT101, TissueScan, Breast Cancer cDNA Array I), containing 48 samples covering 7-normal, 10- Stage I, 13-IIA, 7-IIB, 8-IIIA, 3-IIIC.

cDNA end-point PCR amplification was performed using JumpStart RedTaq (Sigma). The Applied Biosystems Veriti 96-well thermal cycler (Applied Biosystems) and Gel DOC EZ imager (Bio-Rad) were used for End-point polymerase chain reaction analyses.

Gene expression was quantified by real-time qPCR in QuantStudio 6 instrument (Applied Biosystems) using PerfeCta SYBR Green FastMix ROX (Quanta Biosciences) and specific oligonucleotide primers (Table 1). The reaction mixtures contained 10 μl PerfeCta SYBR Green FastMix, 7.2 μl ddH2O, 2.0 μl template cDNA and 0.4 μl gene-specific 10 μM PCR oligonucleotides primers. The reaction conditions were 95°C for 30 s, followed by 40 cycles of 95°C for 5 s and 60°C for 30 s and Melt Curve (dissociation stage). Relative gene expression was calculated as delta (Δ Re (the difference between the cycle threshold values, Ct, of the internal control, and Ct of gene of interest) and confirmed by 2–ΔΔ CT method [67]. In TissueScan samples the non-detects where replaced with the maximum CT value. Due to this replacement the only conclusion extrapolated was expression vs. non expression.

**Table 1:** Real time qPCR primers used for the study

	Forward primer	Reverse primer
GSP-Aromatase		AACAAGGCCGGGGCCTGACA
GSP-Actin		AGCACTGTGTTGGCGTACAG
Ex II	GGGATCGGCAGTGCCTGCAA	AACAAGGCCGGGGCCTGACA
Beta actin	GGACTTCGAGCAAGAGATGG	AGCACTGTGTTGGCGTACAG
I.1 promoter	TCCTATCAGGACGGAAGGTC	CCAAGAGAAAAAGGCCAGTG
2a promoter	GAAAAATCCGCACACACAAA	CCAAGAGAAAAAGGCCAGTG
I.4 promoter	GAGGTCACAGAAGGCAGAGG	GAGGGGGCAATTTAGAGTCC
I.2 promoter	GCAAGCCATGGATTTTGTCT	GAGAAAAAGGCCAGTGAGGA
I.3 promoter	CAAGGTCAGAAATGCTGCAA	GCACGATGCTGGTGATGTTA
PII promoter	CTGCTCCTCACTGGCCTTTT	CATCCACAGGAATCTGCCGT

### Nuclear and cytoplasmic extraction

The breast cells used for nuclear and cytoplasmic fractionation were MCF7, MDA-MB-231, MDA-MB-468 and BT-549 cells. 8 × 10^6^ cells were seeded in a 150mm dish and the cells were allowed to grow until they reached 70% confluency. Nuclear and cytosolic extracts were prepared using NE-PER kit (Thermo Scientific). Cytosolic and nuclear extracts were quantified using BCA reagents (Thermo Scientific) and 50μg of protein was used for Western blotting.

### Western blots

Nuclear and cytoplasmic extracts were subjected to polyacrylamide gel electrophoresis using 4-12% Bolt gel system (Invitrogen), transferred to PVDF (Millipore) membranes, and immunoblotted. Antibodies used in Western blot are as follows: DVL-1 (D3570; Sigma), DVL-3 (SAB4200007; Sigma), Lamin (CS-4777; Cell Signaling), Aromatase (124776; Abcam), GAPDH (sc-47724; Santa Cruz Biotechnology, Inc) and β-actin (sc-47778; Santa Cruz Biotechnology, Inc). Membranes were incubated in 5% milk dissolved in TBST with primary antibody overnight at 4°C. Membranes were washed three time for 10 minutes each with TBST and probed with horseradish peroxidase-conjugated secondary antibodies in 5% milk/TBST for 1 hour at room temperature. Membranes were washed with TBST as previously described before visualization by enhanced chemiluminescence reagent (Thermo Scientific) on premium X-ray films (Phenix Research).

### Immunofluorescence

8 × 10^5^ cells were seeded onto coverslips (12mm) in a 60mm tissue culture dish. The cells were fixed with 4% paraformaldehyde for 15 minutes at room temperature, followed by a wash with PBS for 5 minutes, a quench step with 50mM ammonium chloride (NH_4_Cl) in PBS for 5 minutes with an additional 5 minutes PBS wash. The coverslips were blocked with 5% Bovine serum albumin (BSA) in PBS (blocking buffer) for 30 minutes, followed by an hour incubation with the following primary antibodies in 5% BSA in PBS: DVL-1 (D3570; Sigma) and DVL-3 (SAB4200007; Sigma). The samples were rinsed 3 times with PBS and then incubated with secondary antibodies purchased from ThermoFisher scientific (Alexa flour 568 #A11036, Alexa fluor 647 #A21235 and Alexa fluor phalloidin 488 #A12379 from Thermo Scientific) for 1 hour at room temperature. The samples were rinsed several times in PBS for 5 minutes each and then mounted with prolong gold antifade mounting solution with DAPI (P36941, Thermo Scientific), then cured overnight at room temperature and stored at −20°C until imaged. The samples were imaged using a laser scanning confocal microscope Nikon T-1E with a 60x objective and NIS software.

### Chromatin immunoprecipitation

Cells were grown to confluence in 150mm dishes; a final count of approximately 10 × 10^6^ cells per plate. Proteins were cross-linked to DNA using formaldehyde (Sigma) added directly to the culture medium at a final concentration of 1% for 8 minutes at room temperature. The cross-linking reaction was quenched by adding glycine (Sigma) to a final concentration of 0.125M for 5 minutes at room temperature. The medium was then removed and the cells were washed twice with 1X PBS containing a protease inhibitor cocktail. Then the cells were scraped, pelleted and washed twice with PBS plus protease inhibitor cocktail as described above. Cells were resuspended in SDS Lysis buffer (50mM Tris-HCl pH 8.0, 10mM 0.5M EDTA, and 1% SDS) with protease inhibitor cocktail. Cells were sonicated in a Diagenode Bioruptor sonicator for 25 cycles (30 second pulses and 30 second rest). The soluble chromatin fraction was quantitated and 100μg of chromatin was incubated overnight at 4°C with DVL-1 (D3570; Sigma), DVL-3 (SAB4200007; Sigma), FoxA1 (ab23738; Abcam), and Rabbit IgG (I5006; Sigma). Next day, 11μl of Dynabeads Protein A (Invitrogen) was added to the chromatin-antibody mixture and incubated with rotation for 2.5 hours at 4°C. ChIPs were washed with five low salt wash buffer (0.1% SDS, 1% Triton X-100, 2mM EDTA, 20mM Tris HCl pH 8.1, and 150mM NaCl), three high salt wash buffer (0.1% SDS, 1% Triton X-100, 2mM EDTA, 20mM Tris HCl pH 8.1, and 500mM NaCl), and one TE wash (1mM EDTA and 10mM Tris HCl pH 8). Crosslinks were reversed overnight at 65°C, followed by RNAseA (Promega) at 37°C for 2 hours, and proteinase K incubation (Promega) at 55°C for 2 hours. DNA was eluted using Qiaquick PCR purification kit (Qiagen) and amplified by PCR (Table 2). DVL binding on I.1 promoter was quantified by real-time qPCR in QuantStudio 6 instrument (Applied Biosystems) using PerfeCta SYBR Green FastMix ROX (Quanta Biosciences) and specific ChIP primers (Table 2). The reaction mixtures contained 10 μl PerfeCta SYBR Green FastMix, 7.2 μl ddH2O, 2.0 μl template cDNA and 0.4 μl gene-specific 10 μM PCR ChIP primers. The reaction conditions were 95°C for 30 s, followed by 40 cycles of 95°C for 5 s and 60°C for 30 s and Melt Curve (dissociation stage). Relative gene expression was calculated as delta (Δ Re (the difference between the cycle threshold values, Ct, of the internal control, and Ct of gene of interest) and confirmed by 2–ΔΔ CT method [67].

**Table 2:** ChIP primers used for the study

Promoter region	Forward primer	Reverse primer
I.1 (-533)	TCACCCCCAACACATAGCAC	CCACCACACACCACATTGTTC
I.1 (-802)	GAGGGAGGGTTGACACTCAG	CCAGCTGCTCACAGGGTAAT
I.1 (-835)	GAGGGAGGGTTGACACTCAG	TGGTGGGTATTGCTGAGAGATG
I.1 (-1149)	GGTAGAGCCTCTGAGAATGCAC	CATCCCCTCCCAGTCATCAT
2a	CAATCAGTGTGATGGCCCCT	GTCAGAAGACACCCCACCAG
I.4	CACTCACCTGGCACCTAACC	AAGAGCCACACACTGGGAAG
pII	CGTCACTCTACCCACTCAAGG	AGTCTCAGGTTCCTTTAGACGC

### DVL stable knock-down

MCF7 and BT-549 were infected by pLKO.1-puro based shRNA MISSION lentiviral transduction particles purchased from Sigma for DVL-1 (TRCN0000441114), DVL-3 (TRCN0000033344, TRCN0000033347) and Non-Targeting shRNA control transduction particles (SHC002V). 24h prior transduction, cells were plated at the seeding density 47.5 × 10^3^ cells/cm^2^ in order to reach 80% confluency at the time of transduction. The transduction was enhanced with Hexadimethrine Bromide (Sigma) at a final concentration of 8 μg/ml. Following the addition of hexadimethrine bromide, the appropriate amount of viral particles were added at 2x multiplicity of infection (MOI) to the media, which was replaced with fresh media after 24h. The puromycin selection was started 72h after transduction at a concentration of 0.5 μg/ml, and the puromycin-containing media was replaced every 3-4 days until total selection was achieved.

### Enzyme-linked immunosorbent assay

Cells were seeded in charcoal stripped serum (ThermoFisher) media and supplemented with 10 nmol/L Androstenedione (Sigma). Supernatant culture media were evaluated for estrogen production using estradiol ELISA Kit (Cayman Chemical) following the manufacturer's protocol at 412 nm absorbance using an Infinite M100 PRO Quadruple monochromator microplate reader (Tecan). Standard curve and estradiol concentrations were determined with Cayman ELISA competitive analysis tool.

### Proliferation assay

Cells were seeded in a 96 well plate at 2.5×10^4^ per well and the plates were added to IncuCyte ZOOM Live-Cell Analysis System (Essen Bioscience). The IncuCyte system was capturing 4 phase-contrast images per well every 2 hours to construct the growth curves from confluence measurements of images.

### Statistical analysis

Statistical analysis was performed using unpaired Student's t tests (Graph Pad Prism software) to assess whether differences observed in the various experiments were significant. All results are expressed as mean ± SEM and considered significant at ^*^ p<0.05, ^**^ p<0.01 and ^***^ p<0.001.

## SUPPLEMENTARY MATERIALS FIGURES



## Abbreviations

DVL: Dishevelled
E_2_: 17-β-estradiol
Aromatase: CYP19A1
BMP4: Bone Morphogenetic Protein 4
FZD: Frizzled class receptor
FOXK: Forkhead box K
NSCLC: Non-small cell lung cancer
SNP: Single nucleotide polymorphism
AI: Aromatase inhibitor
SIRT-1: Sirtuin 1
ER: Estrogen receptor
UTR: Untranslated region
PR: Progesterone receptor
HER2: Human epidermal growth factor receptor 2
TIAM1: T cell lymphoma invasion and metastasis 1
FOXA1: Forkhead box A1
NTC: Non-targeting control
LC-MS/MS: Liquid chromatography tandem-mass spectrometry
PTM: Post-translational modification
