# Supplementary material for: DVL1 and DVL3 differentially localize to CYP19A1 promoters and regulate aromatase mRNA in breast cancer cells
Source: Oncotarget. 2018 Nov 2;9(86):35639–54. doi: 10.18632/oncotarget.26257 (PMC6235026; doi:10.18632/oncotarget.26257)
Supplement: Supplementary file 1 [file oncotarget-09-35639-s001.pdf]

DVL1 and DVL3 differentially localize to CYP19A1 promoters and regulate aromatase mRNA in breast cancer cells

SUPPLEMENTARY MATERIALS

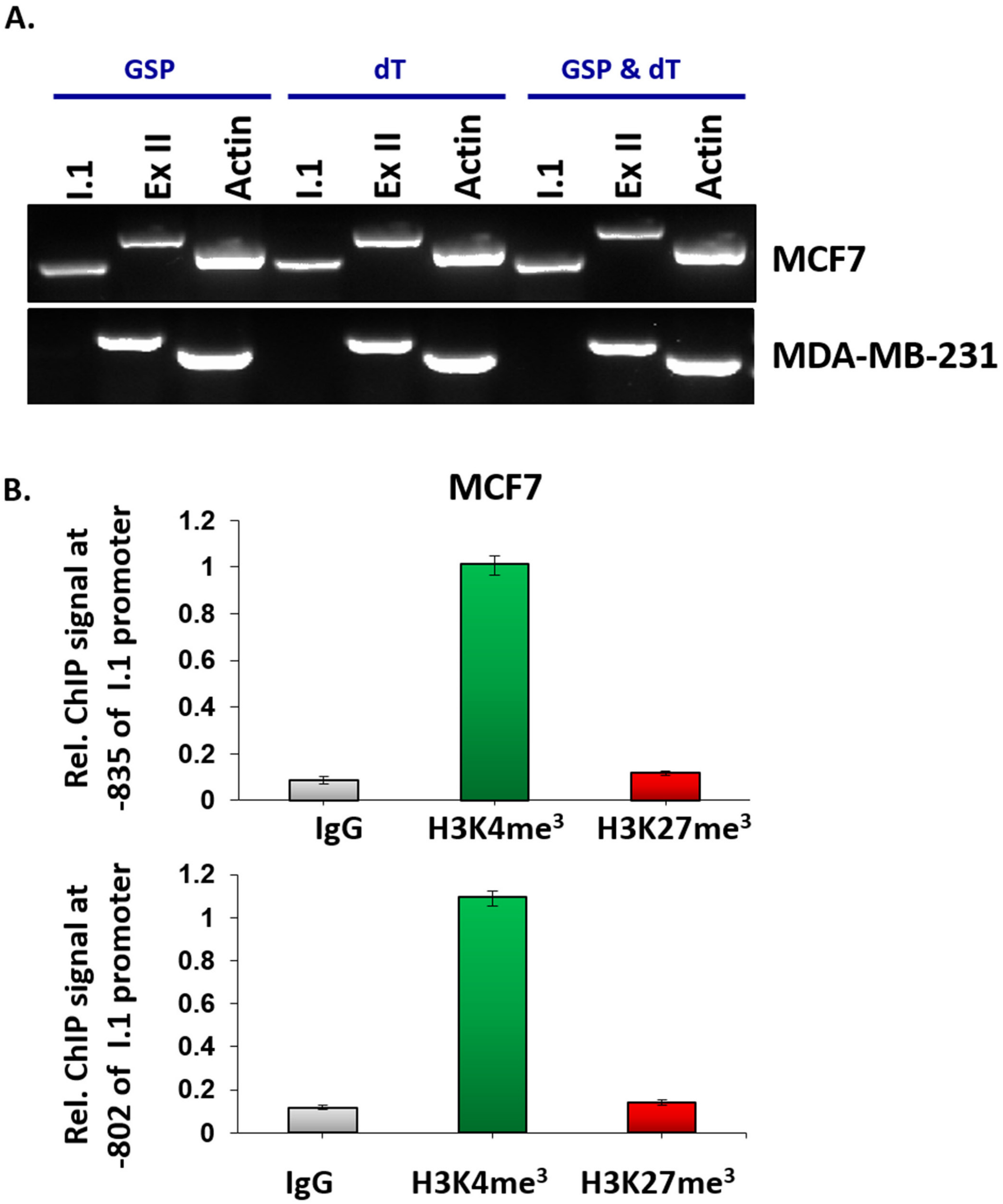

Supplementary Figure 1: The I. 1 transcript is expressed in MCF7 cells. (A) Identification of aromatase active promoters in MCF7 by end-point PCR. Amplification of the placental aromatase was performed using cDNA generated with a gene specific primer to aromatase (GSP), oligo dT (dT) or a combination of both (GSP & dT). To amplify actin, a GSP for actin was included simultaneously with an aromatase GSP. **(B)** ChIP was performed in MCF7 cells at the I.1 promoter. Identification of active mark (H3K4me3) or repressive mark (H3K27me3) by ChIP-qPCR in the region -835 and -802 upstream of the TSS for the I.1 exon, represented as relative ChIP signal respect to the Input.

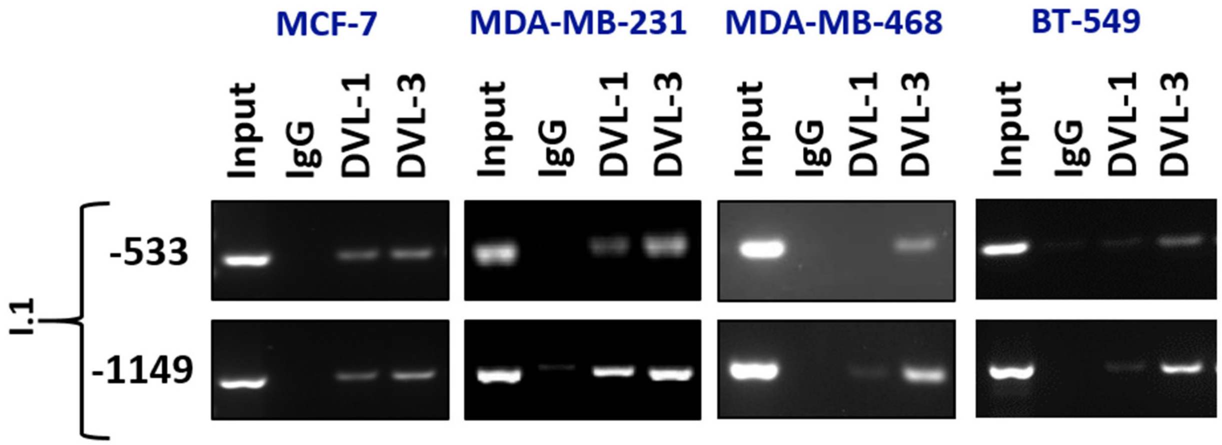

Supplementary Figure 2: DVL family members bind to multiple CYP19A1 promoters. Multiple independent ChIP experiments for IgG, DVL-1, and DVL-3 were performed in MCF7, MDA-MB-231, MDA-MB-468 and BT-549 cells. Occupancy of DVL at the placental I.1 promoter of the CYP19A1 gene was analyzed by end-point PCR.
